# Reninoma in an adolescent boy with negative selective renal vein sampling: a case report and review of the literature

**DOI:** 10.3389/fendo.2026.1942257

**Published:** 2026-09-17

**Authors:** Lidan Zhang, Dongxu Qiu, Jia Li, Xiaoyu Ma, Yuan Xiao, Zhiya Dong, Yushi Zhang, Limin Zhu, Zhenwei Xia, Wenli Lu

**Affiliations:** 1 Department of Pediatrics, Ruijin Hospital, Shanghai Jiao Tong University School of Medicine, Shanghai, China; 2 Department of Urology, Peking Union Medical College Hospital, Chinese Academy of Medical Sciences and Peking Union Medical College, Beijing, China; 3 Department of Hypertension, Ruijin Hospital, Shanghai Jiao Tong University School of Medicine, Shanghai, China

**Keywords:** case report, contrast-enhanced ultrasound (CEUS), juxtaglomerular cell tumor, reninoma, secondary hypertension, selective renal vein sampling

## Abstract

Juxtaglomerular cell tumor (reninoma) is a rare benign renal neoplasm causing secondary hypertension via autonomous renin over-secretion, which is extremely rare in children and adolescents. Here we report a 13-year-old boy with a two-year misdiagnosis of resistant hypokalemic hypertension, who underwent unnecessary left adrenalectomy based on ambiguous adrenal computed tomography (CT). Biochemical tests revealed hyperreninemic hyperaldosteronism with normal aldosterone-to-renin ratio. Renal artery CT identified a tiny low-density lesion in the upper pole of the left kidney, while contrast-enhanced ultrasound (CEUS) clearly displayed characteristic hypoperfusion of the mass. Selective renal vein sampling (SRVS) failed to demonstrate a lateralized renin gradient, a finding consistent with the known low sensitivity of this test for small juxtaglomerular cell tumors (JGCT). Laparoscopic nephron-sparing tumorectomy was performed, and histopathological and clinical features together supported the diagnosis of reninoma. Blood pressure and serum electrolytes normalized postoperatively without antihypertensive or potassium supplementation, with complete quantitative biochemical and imaging surveillance confirming stable remission over the 3-year follow-up. This case highlights that CEUS acts as a radiation-free auxiliary localization modality for suspected pediatric reninoma and provides complementary functional information when SRVS yields false-negative results; definitive diagnosis requires integrated assessment of clinical manifestations, biochemical markers, multimodal imaging, and pathological findings rather than relying on a single imaging technique. Clinicians should raise vigilance of reninoma in children with refractory hypokalemic hypertension to avoid misdiagnosis and unnecessary adrenal surgery.

## 1 Introduction

Hypertension in children and adolescents always requires systematic screening for secondary etiologies, including renal parenchymal lesions, renovascular stenosis, and endocrine neoplasms (1). Juxtaglomerular cell tumor (JGCT), also termed reninoma, is a rare benign renal tumor first described in 1967 (2). Tumor cells constitutively secrete renin to activate the renin-angiotensin-aldosterone system (RAAS), resulting in the classic triad of hypertension, hypokalemia, and elevated plasma renin and aldosterone levels.

Globally, fewer than 200 reninoma cases have been documented (3). Most patients are young females (4). Nonspecific symptoms such as headache and fatigue, combined with the small size of most lesions, frequently lead to delayed diagnosis (5). Selective renal vein sampling (SRVS) has long been regarded as the gold standard for lateralizing renin-secreting lesions, yet false-negative findings are rarely reported in existing literature. In this report, we describe an adolescent male patient with a two-year history of misdiagnosis and unnecessary adrenalectomy, who presented negative SRVS results but definitive diagnostic findings on contrast-enhanced ultrasound (CEUS) (6), and finally received successful nephron-sparing surgery (7). We further review relevant literature to summarize common diagnostic pitfalls and highlight the auxiliary preoperative localization value of radiation-free CEUS as a complementary modality in pediatric reninoma, providing practical clinical strategies for pediatric endocrinologists and radiologists.

## 2 Case description

### 2.1 Patient information

A 13-year-10-month-old male adolescent student was enrolled with a chief complaint of recurrent cephalalgia and bilateral lower-extremity asthenia complicated by uncontrolled severe hypertension persisting for 2 years, with peak blood pressure reaching 188/122 mmHg (1). Anthropometric parameters included height 153 cm and body mass index 23.53 kg/m². No familial history of early-onset hypertension, hereditary renal neoplasia, or monogenic hypertensive disorders was identified.

The patient received seven admissions at local tertiary institutions, managed with chronic oral potassium supplementation and combined angiotensin-converting enzyme inhibitor/angiotensin II receptor blocker antihypertensive regimens. Abdominal computed tomography incidentally identified a suspected unilateral adrenal lesion, prompting left adrenalectomy; nevertheless, hypertensive episodes and hypokalemia persisted without clinical amelioration postoperatively. No chronic comorbidities, occupational toxicant exposure, or developmental abnormalities were documented.

### 2.2 Clinical findings

Admission vital sign assessment recorded bilateral upper-extremity blood pressure of 169/108 mmHg, heart rate 88 beats/min, respiratory rate 18 breaths/min, body temperature 36.7 °C, and oxygen saturation 98% on room air. General examination revealed mild overweight as the sole positive finding. No facial edema, skin pallor, café-au-lait spots, or thyroid enlargement was noted. Cardiac auscultation revealed regular rhythm with normal S1/S2 and no murmurs, gallops, or pericardial rubs. Pulmonary examination showed clear breath sounds bilaterally without rales, wheezes, or diminished breath sounds. Abdominal examination revealed a soft, non-tender abdomen without palpable masses, organomegaly, or abdominal bruits; no costovertebral angle tenderness was elicited. Neurological examination demonstrated normal muscle strength and tone in all four limbs, symmetric deep tendon reflexes, and negative Babinski signs. Fundoscopic examination showed no papilledema, hemorrhages, exudates, or arteriovenous nicking.

### 2.3 Diagnostic timeline

**Table 1** chronologically enumerates the complete diagnostic and therapeutic workflow throughout the disease course.

**Table 1:** Diagnostic and therapeutic timeline of the patient.

Time node	Core clinical events
Initial 2 years post-symptom onset	Recurrent headache, fatigue, refractory hypokalemic hypertension; repeated hospitalizations for antihypertensive and potassium-repletion therapy
Pre-adrenalectomy at local hospital	Abdominal CT suspected adrenal pathology; total left adrenal resection performed
Post-adrenalectomy interval	Sustained hypertension and hypokalemia without remission; interhospital referral to our institution for definitive evaluation
Index admission to our center	Comprehensive laboratory testing including plasma renin activity, aldosterone, captopril provocation test, and renal artery CTA
Advanced localization workup	CEUS and segmental bilateral SRVS
Post-definitive diagnosis	Laparoscopic nephron-sparing enucleation of left renal juxtaglomerular cell tumor
Postoperative Months 1, 3, 6, 12, 24, 36	Serial surveillance of blood pressure, serum electrolytes, and RAAS biomarkers; absence of radiological tumor recurrence

### 2.4 Diagnostic assessment

Biochemical profiling demonstrated the canonical laboratory signature of reninoma. At the first admission (prior to antihypertensive and potassium-supplement therapy), laboratory tests showed hypokalemia with serum potassium of 2.5 mmol/L (reference 3.5–5.3 mmol/L). Baseline plasma renin activity (PRA) was markedly elevated at 8.46 μg/L/h (reference 0.13–1.94 μg/L/h), and plasma aldosterone (ALD) was 242 ng/L (reference 30–170 ng/L). The captopril challenge test confirmed persistent RAAS hyperactivation (**Table 2**). After initiation of combination therapy including valsartan, amlodipine, spironolactone and oral potassium supplementation, the patient was re-evaluated at half-year follow-up. Follow-up assays revealed sustained elevation of PRA and aldosterone, with serum potassium partially corrected to 3.22 mmol/L. Germline genetic sequencing for hereditary secondary hypertension returned negative, excluding monogenic hypertensive syndromes.

**Table 2:** Biochemical characteristics of the patient at different time points.

			First admission (untreated)		6-month follow-up (on treatment)		Captopril test (25 mg oral)		
Variable	Unit	Reference range	Supine	Upright	Baseline	Upright	Supine	Pre-dose	Post-dose
PRA	ng/mL/h	0.1–5.5 (supine) 0.7–17.4 (upright)	8.46	11.70	6.13	7.44	>12	>12	>12
Angiotensin II	pg/mL	16.2–64.2	70.51	88.01	128.62	124.83	269.09	327.47	117.83
Aldosterone	pg/mL	29–161 (supine) 38.1–313.3 (upright)	242.0	391.7	554	1211	172.6	184.9	165.6
24-h urinary aldosterone	μg/24 h	2.25–21.4	—	—	44.92	44.92	—	—	—
Serum potassium	mmol/L	3.5–5.5	2.5	—	3.22		—	—	—
Blood pressure	mmHg	<130/80	—	—	133/92	—	—	—	—

PRA, plasma renin activity; —, not measured.

Values above the reference range are presented without special marking; the reference range for each assay is provided in the third column.

Initial medication (after first admission): amlodipine 5 mg once daily, valsartan 80 mg once daily, spironolactone 20 mg twice daily, potassium chloride sustained-release 0.5 g three times daily.Blood pressure at initial treatment phase: 103–146/59–103 mmHg; serum potassium: 3.8 mmol/L.

Medication at 6-month follow-up: amlodipine 10 mg once daily, valsartan 80 mg once daily, spironolactone 20 mg twice daily.

Multimodal imaging exhibited discordant localization results. Renal artery computed tomography angiography (CTA) detected a 10 × 9 mm hypodense nodule at the left renal upper pole without pathognomonic perfusion features (**Figures 1A, B**). Contrast-enhanced ultrasound (CEUS) revealed characteristic reninoma enhancement kinetics: synchronous cortical arterial-phase opacification with peak signal intensity inferior to adjacent healthy renal parenchyma, demarcating clear tumor margins (**Figures 1C, D**). Segmental venous sampling from bilateral renal veins and the inferior vena cava demonstrated no lateralized renin gradient(**Table 3**), a finding consistent with the known low sensitivity of SRVS for small juxtaglomerular cell tumors and therefore not excluding the diagnosis.

**Figure 1:**
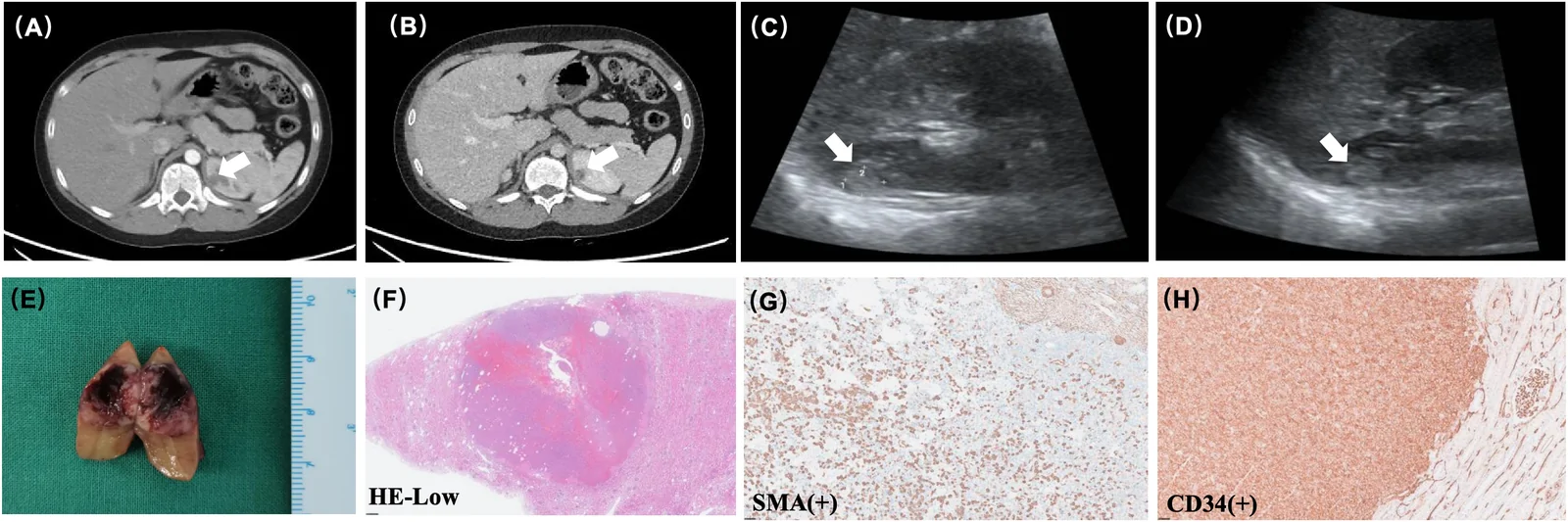
Radiological, gross pathological and histopathological features of renal juxtaglomerular cell tumor in an adolescent patient. **(A, B)** Axial renal artery CTA shows a small hypodense nodule located at the upper pole of the left kidney (white arrow). **(C, D)** Contrast-enhanced ultrasound demonstrates the lesion with typical perfusion characteristics of reninoma. **(E)** Gross appearance of the completely resected subcapsular renal tumor. **(F)** Hematoxylin-eosin staining shows tumor surrounded by normal renal parenchyma. **(G, H)** Immunohistochemical staining for SMA and CD34 shows diffuse positive expression in tumor cells.

**Table 3:** The results of renal venous sampling.

Sampling site	PRA ng/mL/h	AT-II pg/ml	ALD ng/dl
Peripheral blood vessels	>12	530.2	27.82
Proximal end of inferior vena cava	>12	382.7	31.14
Distal end of inferior vena cava	>12	399.5	30.48
Left renal vein	>12	499.9	30.01
Right renal vein	>12	183	26.65

### 2.5 Therapeutic intervention

Preoperative medical optimization consisted of standardized oral potassium repletion and titrated antihypertensive agents to stabilize baseline hemodynamics. Minimally invasive laparoscopic nephron-sparing tumorectomy was executed for complete enucleation of the 1.5 cm subcapsular renal mass(**Figure 1E**). Nephron preservation was prioritized in this adolescent patient to preserve long-term renal functional reserve, with no intra-operative complications encountered.

### 2.6 Follow-up and outcomes

All antihypertensive and potassium-supplementing pharmacotherapies were permanently discontinued within days of surgery. Serum potassium and ambulatory blood pressure normalized rapidly in the early postoperative period. Serial quantitative surveillance was performed at 3-, 6-, 12-, 24-, and 36-month postoperative time-points: blood pressure ranged 104-118/64-74 mmHg, serum potassium 4.1-4.4 mmol/L, plasma renin activity 0.38-0.77ng/mL/h, and aldosterone 48-76 pg/mL, all within institutional reference ranges. Glomerular filtration function remained preserved, and no radiological evidence of tumor recurrence was detected on imaging. No long-term surgery-related adverse events were identified throughout the 3-year follow-up.

### 2.7 Histopathological confirmation

Gross pathological inspection identified a well-circumscribed solid renal nodule. Light microscopy revealed the characteristic histomorphology of juxtaglomerular cell tumor. Immunohistochemical staining exhibited diffuse positivity for SMA and CD34 (**Figures 1F–H**), with negative immunoreactivity for AE1/AE3, HMB45, and S-100. This immunophenotype, in combination with typical morphological features and the patient’s clinical-biochemical profile, supports the diagnosis of reninoma, rather than providing definitive proof alone. Unfortunately, no adequate archived residual tumor tissue remains to perform renin immunohistochemistry, which is regarded as the most specific biomarker for juxtaglomerular cell tumor; this represents a limitation of the present case.

## 3 Discussion

Juxtaglomerular cell tumor (reninoma) is an uncommon cause of secondary hypertension in pediatric and adolescent populations, and its clinical identification is often hindered by nonspecific symptoms and tiny tumor size, as demonstrated in the present case. This 13-year-old male patient endured a two-year diagnostic delay and received an unnecessary adrenalectomy caused by misinterpretation of adrenal imaging findings and incomplete preoperative endocrine work-up. Imaging examinations at the outside hospital revealed mild thickening of the left adrenal junction with a focal nodule on contrast-enhanced adrenal CT, as well as a hypoechoic nodule in the left adrenal region on adrenal ultrasound, suggestive of possible adrenal cortical hyperplasia. Owing to these imaging findings, comprehensive endocrine evaluation was not completed before surgery. Notably, plasma renin activity, aldosterone, and full-panel adrenal hormone assessments were not performed prior to adrenal resection at the local hospital. This reflects a critical clinical pitfall: clinicians tend to prioritize adrenal lesions when evaluating adolescents with hypokalemic hypertension while ignoring reninoma as a key differential diagnosis, and surgical intervention should not be undertaken without complete biochemical screening for secondary hypertension etiologies. Consistent with previous case reports, the patient presented the classic biochemical triad of autonomous renin overproduction: refractory hypertension, persistent hypokalemia, and elevated plasma renin and aldosterone levels accompanied by a normal aldosterone-to-renin ratio, which shifted our diagnostic focus from adrenal glands to the kidney.

Multimodal imaging and invasive venous sampling yielded discrepant localization results in this case. Although renal artery CTA identified a suspicious small hypodense lesion in the left renal upper pole, it could not provide functional vascular information. In contrast, contrast-enhanced ultrasound (CEUS) involves no ionizing radiation or nephrotoxic contrast medium and can display the characteristic hypoperfusion sign of reninoma to assist preoperative localization, offering useful complementary benefits for juvenile patients who require repeated examinations rather than representing a superior standalone diagnostic tool. Notably, selective renal vein sampling (SRVS), generally regarded as a useful modality for lateralizing renin-secreting lesions (8), returned a false-negative result without an interrenal renin gradient. As reported by Wong et al (3). SRVS demonstrates relatively low sensitivity for localizing small reninomas, so a non-lateralized gradient does not exclude the diagnosis. Three plausible mechanisms explain this false-negative finding: aberrant peripheral venous drainage of microtumors bypassing the main renal venous trunks, dilution of tumoral renin by large-volume blood flow within renal veins, and intermittent pulsatile renin secretion by small subcapsular lesions. This finding indicates that a negative SRVS result cannot rule out reninoma, and dynamic functional imaging such as CEUS may serve as a helpful complementary tool when imaging and venous sampling results conflict.

Existing literature indicates that reninoma predominantly occurs in young females (9). Few published studies have described patients who underwent redundant adrenal surgery due to misdiagnosis, and systematic analyses of false-negative SRVS among pediatric reninoma patients are scarce. Prior research regarding CEUS mainly focuses on intraoperative guidance for radiofrequency ablation, whereas our work expands its clinical value for preoperative localization in juvenile reninoma. Of note, as highlighted by Gupta et al (10), SMA and CD34 immunostaining are not sufficiently specific to confirm juxtaglomerular cell tumor, because these markers can also be detected in renal glomus tumors. Renin immunohistochemistry is currently considered the most specific biomarker for establishing the diagnosis of reninoma. As mentioned above, our case is limited by the unavailability of remaining archived tissue to perform renin immunostaining. In terms of surgical management, radical nephrectomy or partial nephrectomy was widely adopted in early cases. By contrast, laparoscopic nephron-sparing tumorectomy was performed in our patient, achieving complete tumor resection while maximally preserving normal renal parenchyma (11), which is critical for maintaining long-term renal function in adolescents. The patient’s blood pressure and serum electrolytes normalized within days after surgery, and all laboratory indicators remained stable during the 3-year follow-up, verifying the curative efficacy of kidney-sparing surgery for benign reninoma.

Compared with adult cohorts, pediatric and adolescent reninoma poses extra diagnostic challenges owing to low clinical suspicion, non-specific symptoms, and the frequent small size of the tumor. Multiple case series have reported that misdiagnosis and unnecessary surgical procedures are not uncommon before the correct identification of JGCT, and false-negative SRVS results may delay correct localization even among patients with typical biochemical features. Besides SRVS limitations, differential diagnoses including renal glomus tumors and other renin-secreting neoplasms should be kept in mind during both radiological and pathological work-up. Multimodal diagnostic algorithms combining biochemistry, cross-sectional imaging, and functional ultrasound are therefore recommended for suspected cases, rather than relying on any single test result.

Four core clinical takeaways can be drawn from this case. First, reninoma should be routinely included in the differential diagnosis for children and adolescents with unexplained resistant hypokalemic hypertension to avoid unnecessary adrenal surgery. Second, radiation-free CEUS is the preferred localization modality for suspected pediatric reninoma over conventional CT, as it enables superior hemodynamic evaluation of lesions. Third, clinicians should not make diagnostic judgments solely based on SRVS lateralization results; comprehensive assessment combining biochemical markers and multimodal imaging is mandatory. Fourth, laparoscopic nephron-sparing tumorectomy represents the optimal surgical strategy for juvenile reninoma to preserve long-term renal reserve.

Collectively, this rare case of reninoma in an adolescent male with false-negative SRVS intuitively reveals the risk of diagnostic delay and limitations of invasive localization tests. CEUS may serve as a valuable complementary functional tool to assist preoperative lesion localization and support nephron-sparing surgical planning for suspected pediatric reninoma, though it should not be regarded as a standalone confirmative test. Clinicians should maintain a high index of suspicion for this disease and adopt an integrated multimodal diagnostic workflow to reduce misdiagnosis and corresponding iatrogenic injuries in adolescents with hypertension.

## Patient perspective

I was diagnosed with high blood pressure two years ago, and I had to take several antihypertensive pills every day. I often felt dizzy and tired during school classes and physical education, which made me unable to take part in running and ball games with my classmates. I once underwent an adrenal surgery, but my blood pressure did not get better after the operation, and I felt confused and upset about repeated hospital visits and various blood tests and imaging scans. After doctors found the real lesion on my kidney, I received laparoscopic nephron-sparing surgery. The postoperative recovery was much smoother than my previous operation. My dizziness disappeared soon after discharge, and my blood pressure stayed normal without any medicine. Now I can exercise normally and attend all sports activities at school. I feel much healthier and relaxed, and I am really grateful to all medical staff for finding out the real cause and providing minimally invasive kidney-preserving treatment for me.

## Data Availability

The original contributions presented in the study are included in the article/supplementary material. Further inquiries can be directed to the corresponding author/s.
